# LANA-Mediated Recruitment of Host Polycomb Repressive Complexes onto the KSHV Genome during *De Novo* Infection

**DOI:** 10.1371/journal.ppat.1005878

**Published:** 2016-09-08

**Authors:** Zsolt Toth, Bernadett Papp, Kevin Brulois, Youn Jung Choi, Shou-Jiang Gao, Jae U. Jung

**Affiliations:** Department of Molecular Microbiology and Immunology, Keck School of Medicine, University of Southern California, Zilkha Neurogenetic Institute, Los Angeles, California, United States of America; University of North Carolina at Chapel Hill, UNITED STATES

## Abstract

One of the hallmarks of the latent phase of Kaposi’s sarcoma-associated herpesvirus (KSHV) infection is the global repression of lytic viral gene expression. Following *de novo* KSHV infection, the establishment of latency involves the chromatinization of the incoming viral genomes and recruitment of the host Polycomb repressive complexes (PRC1 and PRC2) to the promoters of lytic genes, which is accompanied by the inhibition of lytic genes. However, the mechanism of how PRCs are recruited to the KSHV episome is still unknown. Utilizing a genetic screen of latent genes in the context of KSHV genome, we identified the latency-associated nuclear antigen (LANA) to be responsible for the genome-wide recruitment of PRCs onto the lytic promoters following infection. We found that LANA initially bound to the KSHV genome right after infection and subsequently recruited PRCs onto the viral lytic promoters, thereby repressing lytic gene expression. Furthermore, both the DNA and chromatin binding activities of LANA were required for the binding of LANA to the KSHV promoters, which was necessary for the recruitment of PRC2 to the lytic promoters during *de novo* KSHV infection. Consequently, the LANA-knockout KSHV could not recruit PRCs to its viral genome upon *de novo* infection, resulting in aberrant lytic gene expression and dysregulation of expression of host genes involved in cell cycle and proliferation pathways. In this report, we demonstrate that KSHV LANA recruits host PRCs onto the lytic promoters to suppress lytic gene expression following *de novo* infection.

## Author Summary

Persistent KSHV infection of humans requires the establishment of viral latency in B cells and endothelial cells following primary infection. This involves the spatially and temporally ordered recruitment of host epigenetic factors onto the viral DNA during *de novo* infection, resulting in the genome-wide repression of lytic (but not latent) gene expression. We have previously shown that the host epigenetic repressor Polycomb Repressive Complexes bind to the KSHV genome and play a role in the inhibition of lytic gene expression following *de novo* infection. Using reverse genetics approaches, we identified the latent KSHV protein LANA to be responsible for the recruitment of the Polycomb proteins onto the promoters of lytic genes following *de novo* infection. Importantly, our study revealed a novel role for LANA in KSHV infection. LANA initiates the recruitment of host repressive epigenetic factors onto the incoming viral genome to suppress lytic gene expression, which is ultimately required for the establishment of KSHV latency in the hosts and the development of KSHV-associated cancers.

## Introduction

It is estimated that 15%-20% of human cancers are caused by viral infections [1]. A hallmark of the seven known human tumor viruses is their ability to cause persistent infection of humans. One of these oncoviruses is Kaposi’s sarcoma-associated herpesvirus (KSHV or Human Herpesvirus 8), the etiologic agent of the vascular tumor Kaposi’s sarcoma (KS), and two B cell lymphomas: primary effusion lymphoma and a subset of multicentric Castleman's disease [2,3]. Accumulating evidence suggests that KSHV pathogenesis depends on the latent infection of B cells and lymphatic endothelial cells, but the mechanism by which latency is established following primary infection is still largely unknown.

KSHV has a double-stranded DNA genome of ~165-kb encoding more than 80 genes [4]. The majority of viral genes encode lytic proteins required for viral DNA replication and virion production, and they are expressed in a temporally ordered manner during reactivation. In contrast, during latency the lytic gene expression is repressed and only the latent genes (the latency-associated nuclear antigen (*LANA* or *ORF73*), *v-Cyclin* (*ORF72*), *v-FLIP* (*K13* or *ORF71*), *Kaposin* (*K12*) and viral *miRNAs*) are constitutively expressed from the latency locus of the viral genome [5]. These latent viral products are involved not only in the maintenance of viral latency, but also in growth-transformation and cell cycle-deregulation, both of which can contribute to the development of KSHV-induced cancers [6]. In fact, transgenic mice expressing KSHV latent genes in B cells develop B cell hyperplasia and lymphomas [7]. Of the latent gene products, LANA has a particularly critical role in both viral pathogenesis and the maintenance of latent infection. LANA is a multifunctional viral protein that ensures the concurrent latent replication of viral DNA with the host genome and the segregation of replicated KSHV genomes into the daughter cells during mitosis [8,9]. In addition, LANA can regulate both viral and cellular gene expression by interacting with numerous transcription and epigenetic factors as well as modulating many signaling pathways [10]. These LANA-associated functions have been implicated to play critical roles in the maintenance of latency by inhibiting lytic gene expression and viral pathogenesis by deregulating host signaling pathways.

Recent works have demonstrated that the establishment of KSHV latency involves a dynamic interaction of the viral genome with cellular epigenetic factors during *de novo* infection [11,12,13]. While the KSHV genome is linear and histone-free in the virion, upon infection it becomes circular and is maintained as an episome that adopts a chromatin structure similar to the host chromosomes [14,15]. We and others have demonstrated that different combinations of activating and repressive histone modifications on the viral episome are involved in the regulation of both the latent and lytic gene expression programs of KSHV [13,16,17,18,19]. Recently, we have shown that, following *de novo* infection, the KSHV genome rapidly becomes chromatinized and undergoes different chromatin states [11,12]. The viral genome initially acquires a transcriptionally permissive chromatin structure (euchromatin marked by H3K4me3 and H3K27ac) that allows the expression of a subset of viral lytic genes. Thereafter, transcriptionally repressive chromatin (heterochromatin) forms on the KSHV genome resulting in the repression of lytic gene expression, a crucial step in the establishment of viral latency. Two of the major cellular transcription repressor complexes for the viral heterochromatin formation are the Polycomb Repressive Complex 1 (PRC1) and 2 (PRC2) [12]. PRC2 is composed of three core subunits (EZH2, SUZ12 and EED) that interact with other transcription repressors such as histone deacetylases and DNA methyltransferases to maintain gene silencing [20]. EZH2 functions as a histone methyltransferase within PRC2, catalyzing the trimethylation of lysine 27 on histone H3 (H3K27me3), a hallmark of PRC2 function on chromatin. PRC1 is also a multisubunit complex containing the histone H2A mono-ubiquitin ligases RING1A/B, its regulatory subunit BMI-1 and other accessory factors. PRCs can repress gene expression in many different ways such as blocking the activity of RNA polymerase II and histone methyltransferases involved in transcription activation [21]. We have previously demonstrated that both PRCs are involved in the downregulation of transient lytic gene expression following KSHV infection. We have shown that both PRCs can bind to the entire KSHV episome after 24 hours postinfection (hpi). As with the host genome, PRC1 recruitment depends on the PRC2-mediated pre-deposition of the repressive histone mark H3K27me3 on the viral chromatin [12]. While the recruitment of PRC2 onto its cellular target genes is often mediated by long non-coding RNAs and transcription factors, the mechanism by which PRC2 is initially recruited onto the KSHV genome following *de novo* infection is still unknown [12,22].

In this report, we hypothesized that KSHV latent factors might be involved in the recruitment of the PRC2 complex onto the viral episomes during *de novo* infection. To address this question, we used reverse genetics to generate KSHV knockout (KO) mutants of genes within the latency locus and tested which latent genes were required for the recruitment of PRC2 onto the KSHV genome during *de novo* infection. Our results show that loss of the *LANA* gene completely abolished the recruitment of EZH2 onto lytic promoters during infection. Consequently, levels of H3K27me3 modification and RING1B occupancy on the LANA-deficient viral genomes were drastically reduced and lytic gene expression was increased. Our results indicate that the KSHV LANA hijacks host polycomb proteins to create the heterochromatin structure on the viral genome, which leads to the repression of lytic gene expression and thereby, the establishment of viral latency.

## Results

### Identification of the latent gene required for the recruitment of polycomb proteins onto the KSHV genome during *de novo* infection

**Fig 1:**
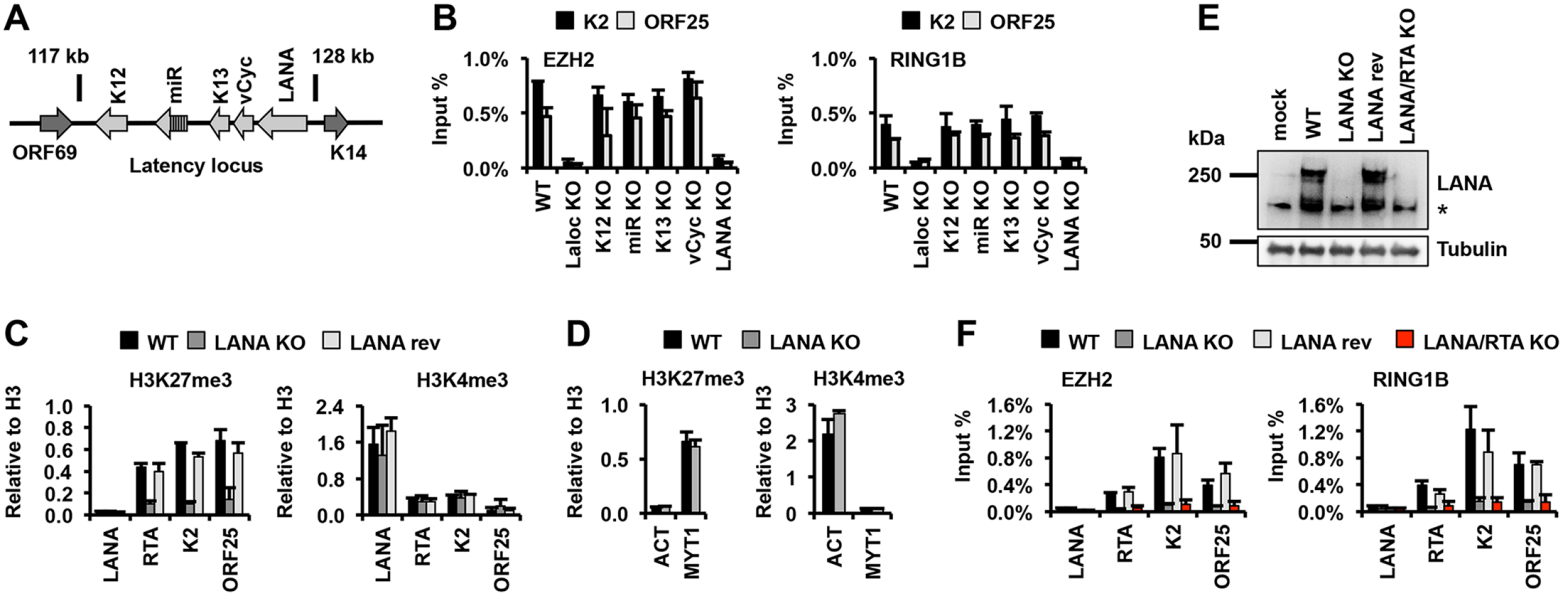
LANA is required for the recruitment of Polycomb proteins to lytic promoters during *de novo* infection. **(A)** Schematic of the KSHV latency locus encoding latent genes. **(B)** ChIP assays testing the recruitment of PRC2 factor EZH2 and PRC1 factor RING1B onto viral lytic promoters in SLK cells 72 hours after infection with WT or the indicated KSHV mutants. **(C and D)** ChIP assays for the repressive (H3K27me3) and activating (H3K4me3) histone marks on viral and cellular promoters at 72 hpi. The LANA and actin (ACT) promoters devoid of H3K27me3 and the PRC2-regulated MYT1 promoter served as controls. **(E)** Immunoblots showing LANA protein levels in mock and KSHV-infected SLK cells at 72 hpi. *Non-specific. **(F)** ChIP assays for EZH2 and RING1B associations with viral promoters at 72 hpi.

To determine which of the latent genes is required for the recruitment of polycomb proteins onto the KSHV genome during *de novo* infection, we generated a series of latent gene KO mutant viruses using en passant mutagenesis on the KSHV BAC clone BAC16 (Fig 1A and S1 Fig). Next, we performed chromatin immunoprecipitation (ChIP) assays for EZH2 and RING1B binding to the promoters of K2 and ORF25 lytic genes in SLK cells infected with WT, latency locus (Laloc) KO or latent gene KO viruses at 72 hpi (Fig 1B). This showed that deletion of either the latency locus or the LANA gene completely abrogated the binding of EZH2 and RING1B to the K2 and ORF25 viral promoters (Fig 1B). Accordingly, at 72hpi, the enrichment of H3K27me3 was greatly reduced on the promoter of RTA, K2 and ORF25 lytic genes in LANA KO KSHV-infected cells compared to WT or LANA revertant (LANA rev) KSHV-infected cells (Fig 1C). In contrast, both the deposition of activating histone mark H3K4me3 on the viral LANA, RTA, and K2 promoters and the occupancy of host transcription factors CTCF and RAD21 on the LANA promoter and in the RTA locus were comparable between WT KSHV- and LANA KO KSHV-infected cells (Fig 1C and S2A Fig). The transcriptionally active promoter of the host actin (ACT) gene and the PRC2-repressed promoter of the host MYT1 gene had comparable occupancy of histone marks H3K27me3 and H3K4me3 in WT KSHV- and LANA KO KSHV-infected cells, indicating the comparable efficiency of ChIPs in both WT and LANA KO-infected cells (Fig 1D). These results indicate a specific role for LANA in the recruitment of EZH2 and thereby, the deposition of H3K27me3 onto the KSHV lytic promoters during *de novo* infection.

LANA has also been implicated in the repression of the KSHV immediate early (IE) gene, RTA, whose expression can induce the dissociation of polycomb proteins from lytic promoters during lytic reactivation [16,23,24]. To exclude the possibility that the loss of EZH2 and RING1B occupancy on viral lytic promoters was due to the expression of RTA in LANA KO KSHV-infected cells, we made a LANA/RTA double KO (dKO) virus (Fig 1E). Our ChIP analysis showed that EZH2 and RING1B recruitment onto the RTA, K2 and ORF25 lytic viral promoters was also abrogated in the LANA/RTA dKO KSHV-infected cells (Fig 1F). Furthermore, shRNA-mediated depletion of LANA expression in SLK cells infected with either WT or RTA KO KSHV also resulted in the reduced deposition of H3K27me3 and RING1B onto lytic promoters at 72 hpi, while the H3K4me3 deposition was not affected (S2B and S2C Fig). Taken together, our results indicate that LANA is required for the recruitment of both PRC1 and PRC2 onto the lytic KSHV promoters during *de novo* infection.

### Genome-wide binding and requirement of LANA for EZH2-binding on the KSHV genome during *de novo* infection

**Fig 2:**
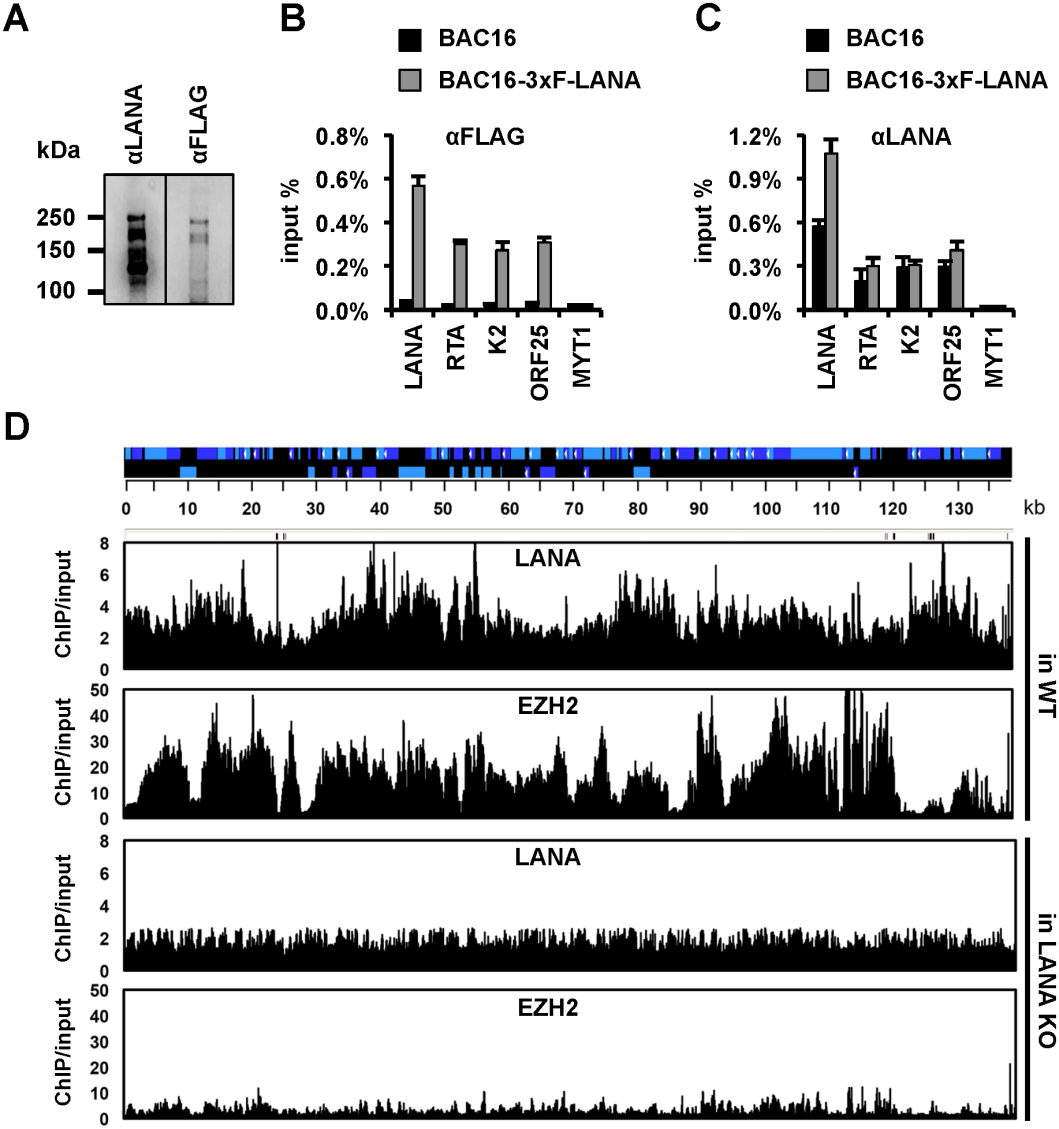
LANA binds to the entire KSHV genome and is required for the recruitment of EZH2 onto the viral episome during *de novo* infection. **(A)** Immunoblot analysis of LANA expression in BAC16-3xF-LANA infected SLK cells at 72 hpi using anti-LANA and anti-FLAG antibodies. **(B and C)** FLAG and LANA ChIP assays for testing LANA-binding to viral promoters in BAC16 and BAC16-3xF-LANA KSHV-infected SLK cells at 72 hpi. Cellular MYT1 promoter was used as a control. **(D)** ChIP-on-chip analysis of the genome-wide binding of LANA and EZH2 to the KSHV genome in WT and LANA KO KSHV-infected cells at 72 hpi.

The genome-wide recruitment of EZH2 onto the KSHV episome during *de novo* infection raises the possibility that LANA binds throughout the entire viral genome to recruit EZH2 [12]. In order to test LANA-binding to the viral episomes during *de novo* infection, we generated recombinant KSHV BAC16-3xF-LANA carrying a N-terminal 3xFLAG tagged LANA (Fig 2A) and used either anti-Flag antibody or anti-LANA antibody to detect LANA-binding to the KSHV genome during *de novo* infection (Fig 2A–2C). ChIP assay with an anti-FLAG antibody or an anti-LANA antibody showed LANA-binding on the EZH2-regulated promoters of KSHV lytic genes RTA, K2 and ORF25 at 72 hpi. The LANA promoter, which possesses a LANA-binding site, and the host MYT1 promoter were used as positive and negative controls, respectively [25]. In order to test if LANA bound throughout the KSHV genome and whether LANA was required for the genome-wide recruitment of EZH2 onto the viral episome, we infected SLK cells with WT KSHV or LANA KO KSHV, and performed ChIP-on-chip assays for LANA and EZH2 at 72 hpi (Fig 2D). We found that LANA was highly enriched on the majority of the KSHV genome in WT virus-infected cells, but LANA-binding to the viral episome was not detected in LANA KO virus-infected cells (Fig 2D). The ChIP-on-chip experiments also revealed that LANA was distributed throughout the viral genome with detectable enrichments at specific genomic regions. Compared to WT KSHV-infected cells, LANA KO-infected cells showed a drastic reduction of the genome-wide recruitment of EZH2 on the viral genome (Fig 2D). These results indicate a widespread binding of LANA on the KSHV genome during *de novo* infection, which is required for EZH2 recruitment onto the KSHV episome.

### Effect of LANA on viral and host gene expression in KSHV-infected cells

**Fig 3:**
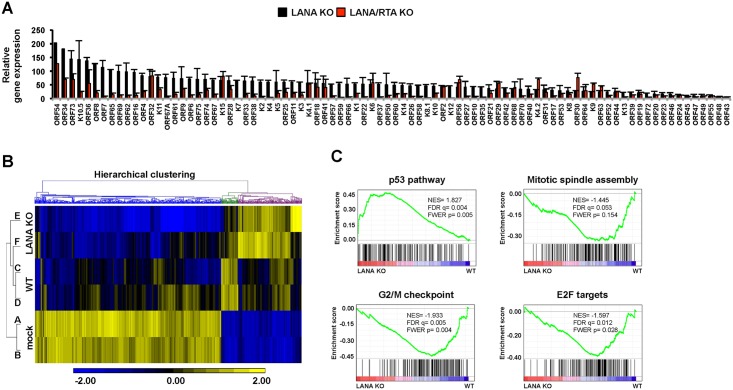
Deregulated viral and cellular gene expression in LANA KO KSHV-infected cells. **(A)** Analysis of viral gene expression in LANA KO and LANA/RTA KO KSHV-infected cells compared to WT KSHV-infected cells by NanoString technology. **(B)** Heat map of differential gene expression between mock and KSHV-infected cells. Rows A and B, C and D, E and F represent two biological replicates of mock-, WT, and LANA KO KSHV-infected SLK cells, respectively. Also see S3 Table. **(C)** Biological pathways significantly affected by the lack of LANA in KSHV-infected cells were determined by gene set enrichment analysis. Also see S4 Table.

LANA has been shown to modulate expression of many host genes involved in the regulation of cell cycle and survival [26]. To determine the impact of LANA on viral and host gene expression during *de novo* infection, we performed NanoString analysis for KSHV gene expression and microarray analysis for host gene expression in WT, LANA KO and LANA/RTA dKO KSHV-infected SLK cells at 72 hpi (Fig 3 and S3 Fig). Our NanoString analysis revealed that the expression of the majority of KSHV genes was highly induced in LANA KO KSHV-infected cells compared to those in WT KSHV-infected cells (Fig 3). However, the expression of most viral genes was drastically reduced in LANA/RTA dKO KSHV-infected cells, suggesting that the lytic genes were upregulated as a result of increased RTA expression in the absence of LANA gene (Fig 3 and S3 Fig). Interestingly, the expression of several viral genes (e.g. ORF29, ORF32, K15, and K6) was comparable in LANA KO KSHV cells *vs*. LANA/RTA dKO KSHV-infected cells, indicating RTA-independent expression of several KSHV genes. The NanoString data were further confirmed by real time quantitative PCR (qPCR) measurement of several viral transcripts (S3B and S3C Fig). In summary, our data show that LANA can function as a genome-wide repressor of lytic gene expression during *de novo* infection.

We performed microarray-based gene expression analyses followed by hierarchical clustering of the microarray datasets to determine differential host gene expression during *de novo* mock infection, WT KSHV infection or LANA KO KSHV infection, (S3 Table, Fig 3B and S3D Fig). This revealed that WT KSHV or LANA KO KSHV infection altered the expression of a number of host genes. The data was organized by hierarchical clustering into three categories: decreased host gene expression upon KSHV infection (blue cluster), increased host gene expression primarily upon WT KSHV infection (green cluster), and increased host gene expression primarily upon LANA KO KSHV infection (purple cluster) (Fig 3B). Gene set enrichment analysis showed that expression of the p53 pathway-related genes was higher in LANA KO virus-infected cells than in WT KSHV-infected cells, whereas expression of the cell cycle-related genes was lower in LANA KO virus-infected cells than in WT KSHV-infected cells (Fig 3C and S4 Table). Furthermore, we asked whether the differences in host gene expression observed between WT virus-infected cells and LANA KO virus-infected cells were solely attributed to the absence of LANA or influenced by the induction of lytic viral gene expression of LANA KO virus. To test this, we used LANA/RTA dKO KSHV, which shows greatly reduced lytic gene expression. Among host genes upregulated in LANA KO virus-infected cells relative to WT virus-infected cells, several were no longer upregulated in LANA/RTA dKO virus-infected cells, indicating that RTA and/or other induced lytic genes can play a role in increased host cell gene expression in LANA KO virus-infected cells during *de novo* infection.

Collectively, these data indicate that LANA is involved in the global inhibition of viral lytic gene expression during *de novo* infection. Furthermore, this inhibition is mainly mediated *via* the repression of RTA expression. In the absence of LANA, increased lytic gene expression affects cell cycle and proliferation pathways following *de novo* infection, which may compromise the establishment of latency.

### LANA-binding precedes the temporally ordered binding of EZH2 on the KSHV genome

**Fig 4:**
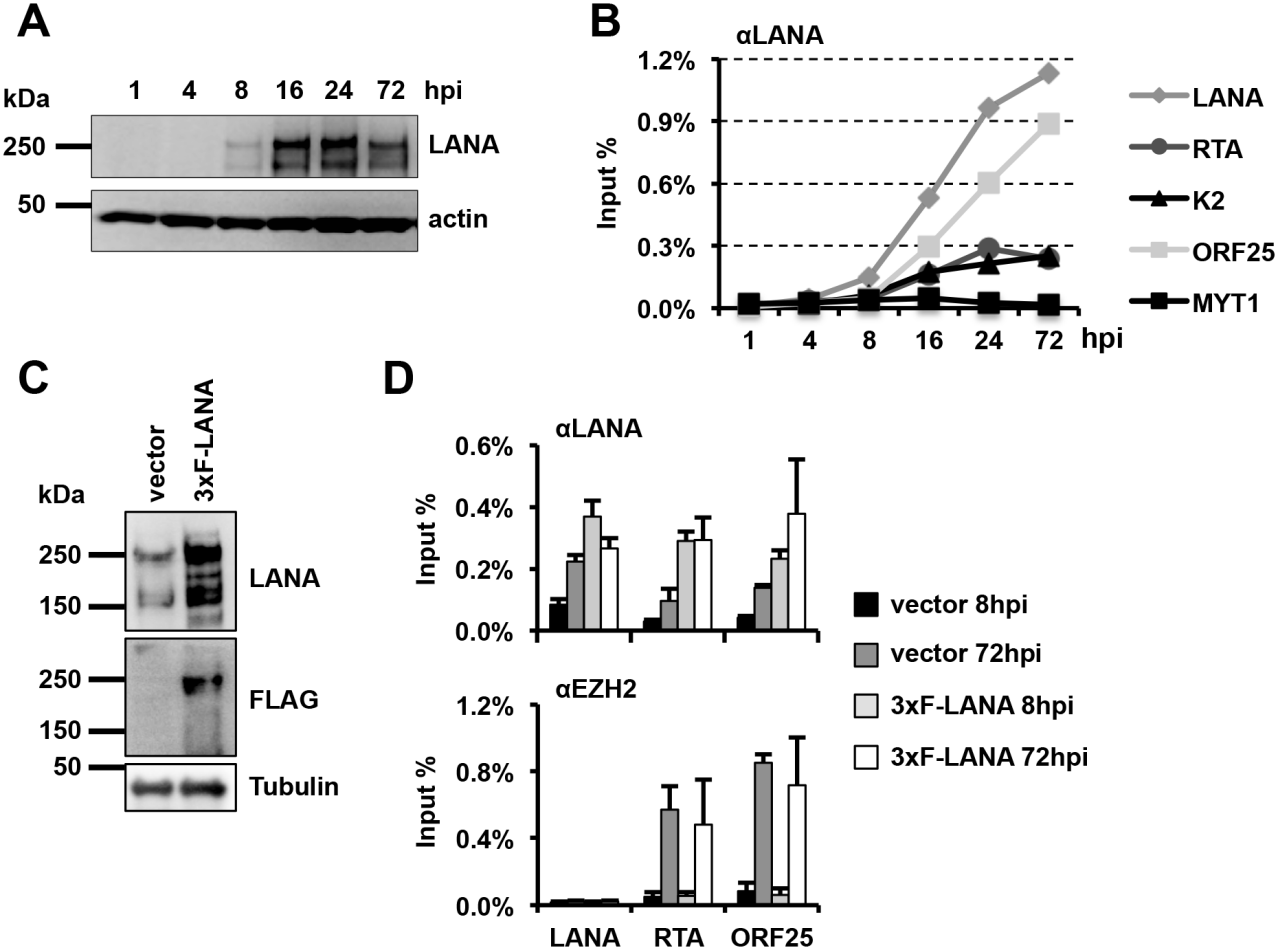
Additional LANA expression is not sufficient for the early recruitment of EZH2 onto the KSHV genome. **(A)** Time course analysis of LANA protein expression during *de novo* infection. **(B)** Time course ChIP analysis of LANA-binding to viral promoters during *de novo* infection. **(C)** Immunoblot analysis of LANA expression in empty lentiviral vector and 3xFLAG-LANA expressing lentivirus infected cells. **(D)** ChIP assays for binding of LANA and EZH2 to viral promoters in LANA-overexpressing cells during *de novo* KSHV infection.

To measure the kinetics of LANA expression and its recruitment to the KSHV genome during *de novo* infection, we infected SLK cells with WT KSHV and analyzed LANA expression by immunoblot and immunofluorescence analyses and the binding of LANA to viral promoters by ChIP assays at 1, 4, 8, 16, 24 and 72 hpi (Fig 4 and S4 Fig). These showed that LANA expression and its recruitment to viral promoters could be detected as early as 8 hpi and further increased thereafter (Figs 4A and 4B and S4A). Immunofluorescence (IF) assay also showed that LANA was detected as puncta in the nucleus of infected SLK cells as early as 8hpi and the number and intensity of puncta increased over the course of infection, indicating a gradual increase of LANA expression and recruitment to viral promoters following KSHV infection (Fig 4B and S4 Fig).

We have previously demonstrated that EZH2 is recruited onto the KSHV genome after 24 hpi [12]. Next, we tested whether ectopic expression of LANA affected the kinetics of EZH2 recruitment onto the KSHV episome before 24 hpi. To address this, we infected SLK cells with lentivirus vector or lentivirus-3xFLAG-LANA followed by WT KSHV infection 24 hours later, and performed ChIPs to test the recruitment of LANA and EZH2 on the promoters of LANA, RTA and ORF25 at 8 and 72 hrs after KSHV infection. Immunoblot analysis with an anti-LANA antibody showed that the LANA level was substantially higher in 3xFLAG-LANA expressing cells than in vector control cells at 8 hrs of KSHV infection (Fig 4C). ChIPs with an anti-LANA antibody also showed higher LANA binding on the viral promoters in 3xFLAG-LANA expressing cells compared to that in vector control cells. In fact, the level of LANA enrichment in 3xFLAG-LANA expressing cells at 8 hpi was comparable or even higher to that on the same viral promoters in vector control cells at 72 hpi (Fig 4D). However, the higher enrichment of LANA on the promoters of lytic genes (RTA and ORF25) resulted in neither faster nor enhanced recruitment of EZH2 in 3xFLAG-LANA expressing cells (Fig 4D). These data indicate that although LANA-binding precedes EZH2 binding on the lytic promoters during *de novo* infection, LANA-binding itself is not sufficient for regulating the temporally ordered recruitment of EZH2 to the KSHV genome.

### Co-localization and enrichment of polycomb proteins with LANA in KSHV infected cells

**Fig 5:**
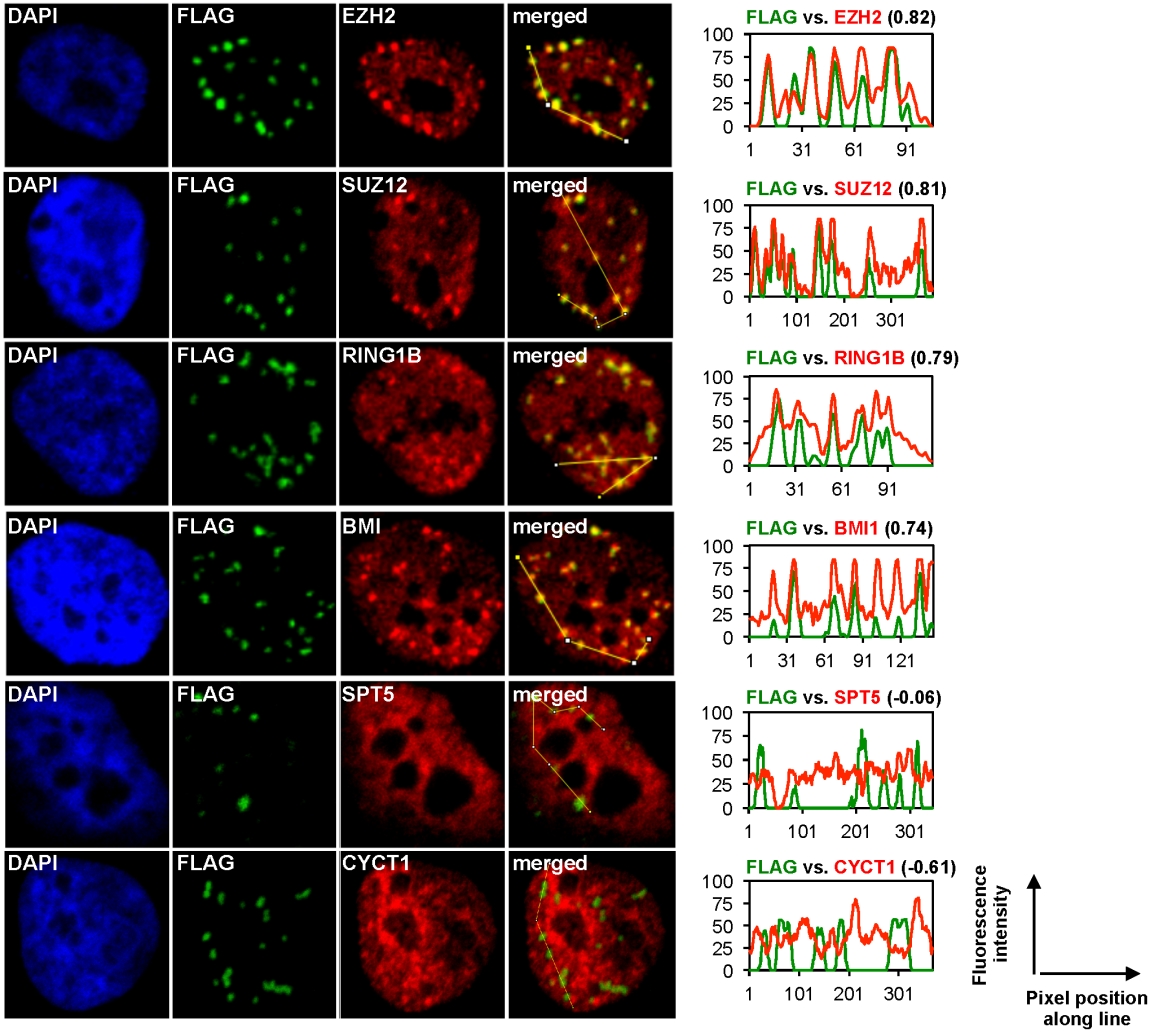
Co-localization and focal enrichment of polycomb proteins with LANA in KSHV-infected cells. KSHV-infected iSLK cells (iSLKBAC16-3xF-LANA) were subjected to confocal microscopic analysis for LANA (Green, false color) and polycomb proteins (Red). SPT5 and CYCT1 served as controls. FLAG antibody was used to detect the 3xF-LANA. Representative LANA puncta were connected by white lines and the co-localization of LANA with the indicated cellular proteins was measured by the image processing program ImageJ.

We investigated whether the LANA-dependent recruitment of polycomb proteins onto the KSHV genome can affect the nuclear localization of polycomb proteins in infected cells. To this end, we performed confocal microscopy for LANA and the components of PRC1 (RING1B and BMI-1) and PRC2 (EZH2 and SUZ12) in *de novo* KSHV infected iSLK cells (96 hpi), reactivated iSLKBAC16, long-term latently infected iSLK cells (iSLKBAC16-3xF-LANA) and TIME cells (TIMEBAC16) (Fig 5 and S5 Fig). The fluorescence signal intensities of LANA (green) and polycomb proteins (red) were measured along marked lines connecting the LANA puncta in the nucleus, and the correlation coefficients of the fluorescence signals were calculated to determine the level of co-localization between LANA and the polycomb proteins. These experiments revealed that while the polycomb proteins were diffused in the nucleus in uninfected cells, they were co-localized and focally enriched with LANA in KSHV-infected cells during *de novo* infection (S5C, S5D and S5K Fig) and latency (Figs 5, S5E, S5F and S5L–S5O). In contrast, KSHV lytic reactivation resulted in the dissociation of LANA and polycomb proteins (S5G and S5H Fig). In addition, the lack of co-localization of LANA with the nuclear factors SPT5 and Cyclin T1 (CycT1) in latently infected cells supports the specificity of LANA co-localization with the polycomb proteins (Fig 5). Interestingly, the colocalization of EZH2 and SUZ12 with LANA could also be observed on the mitotic chromosomes of infected cells, suggesting that LANA and polycomb proteins remain associated through mitosis (S5F and S5M Fig). These results suggest that the focal concentration of LANA and polycomb proteins in the nuclei of infected cells is the result of LANA-mediated recruitment of PRC factors onto the KSHV genome. Strikingly, the co-localization of LANA with the PRC proteins persists through mitosis, suggesting the role of LANA in the maintenance of the PRC-regulated heterochromatin on the KSHV genome in the dividing latently infected cells.

### LANA interacts with PRC2 but not PRC1

**Fig 6:**
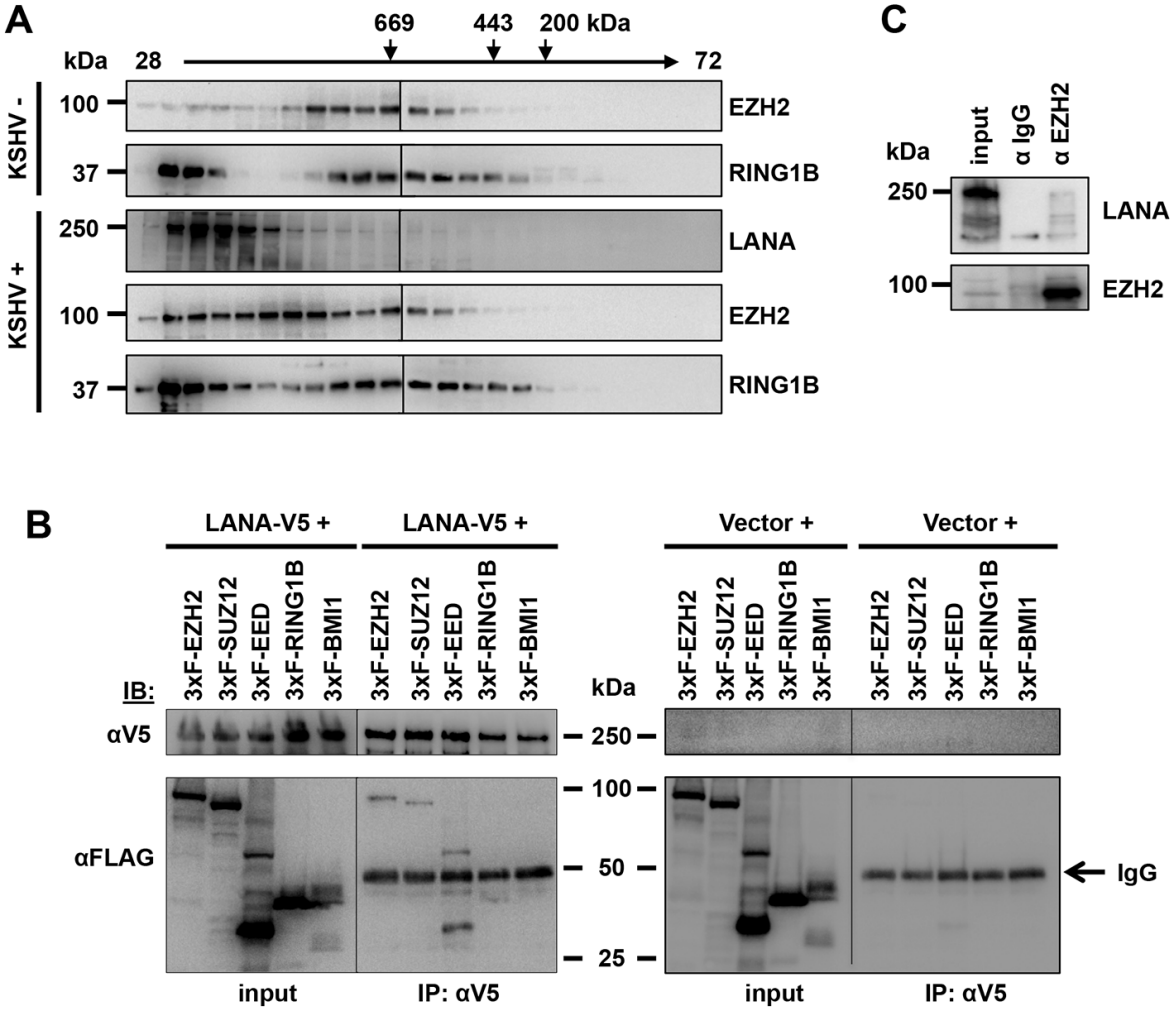
Interaction of LANA with PRC2. **(A)** Gel filtration chromatography of nuclear extracts derived from iSLK and iSLKBAC16 cells. Fractions from 28 to 72 were tested by immunoblot analysis for LANA and polycomb proteins. Molecular weight markers are indicated. **(B)** Co-immunoprecipitation of LANA-V5 (using V5 antibody) from 293T cells expressing LANA-V5 and the indicated 3xFLAG-tagged PRC2 (EZH2, SUZ12 and EED) or PRC1 (RING1B and BMI-1) factors. Immunoprecipitation and input blots were probed with V5 and FLAG antibodies. **(C)** Cell lysates derived from *de novo* KSHV infected iSLK cells (72 hpi) were used for immunoprecipitation with anti-EZH2 rabbit antibody (αEZH2) or normal rabbit IgG (αIgG), followed by immunoblotting with αLANA and αEZH2.

We used gel filtration chromatography and co-immunoprecipitation assays to test whether LANA forms a complex with polycomb proteins in infected cells (Fig 6). Gel filtration assay was performed with nuclear extracts derived from uninfected iSLK and latently KSHV-infected iSLK cells (Fig 6A). This analysis revealed that both EZH2 and RING1B protein levels considerably increased in the LANA-enriched fractions in KSHV-infected cells compared to uninfected cells (fractions 30–38), which is correlated with the co-localization between polycomb proteins and LANA in KSHV-infected cells (Figs 5 and S5). Furthermore, co-immunoprecipitation showed that V5-tagged LANA interacted with 3xFLAG-tagged PRC2 factors (EZH2, SUZ12 or EED) but not with 3xFLAG-tagged PRC1 factors (RING1B or BMI-1) (Fig 6B). Finally, the interaction of LANA with EZH2 was also detected during *de novo* KSHV infection, demonstrating that LANA can form a complex with polycomb proteins in KSHV infected cells (Fig 6C).

### The binding of LANA to the KSHV genome is required for the EZH2 recruitment during *de novo* infection

**Fig 7:**
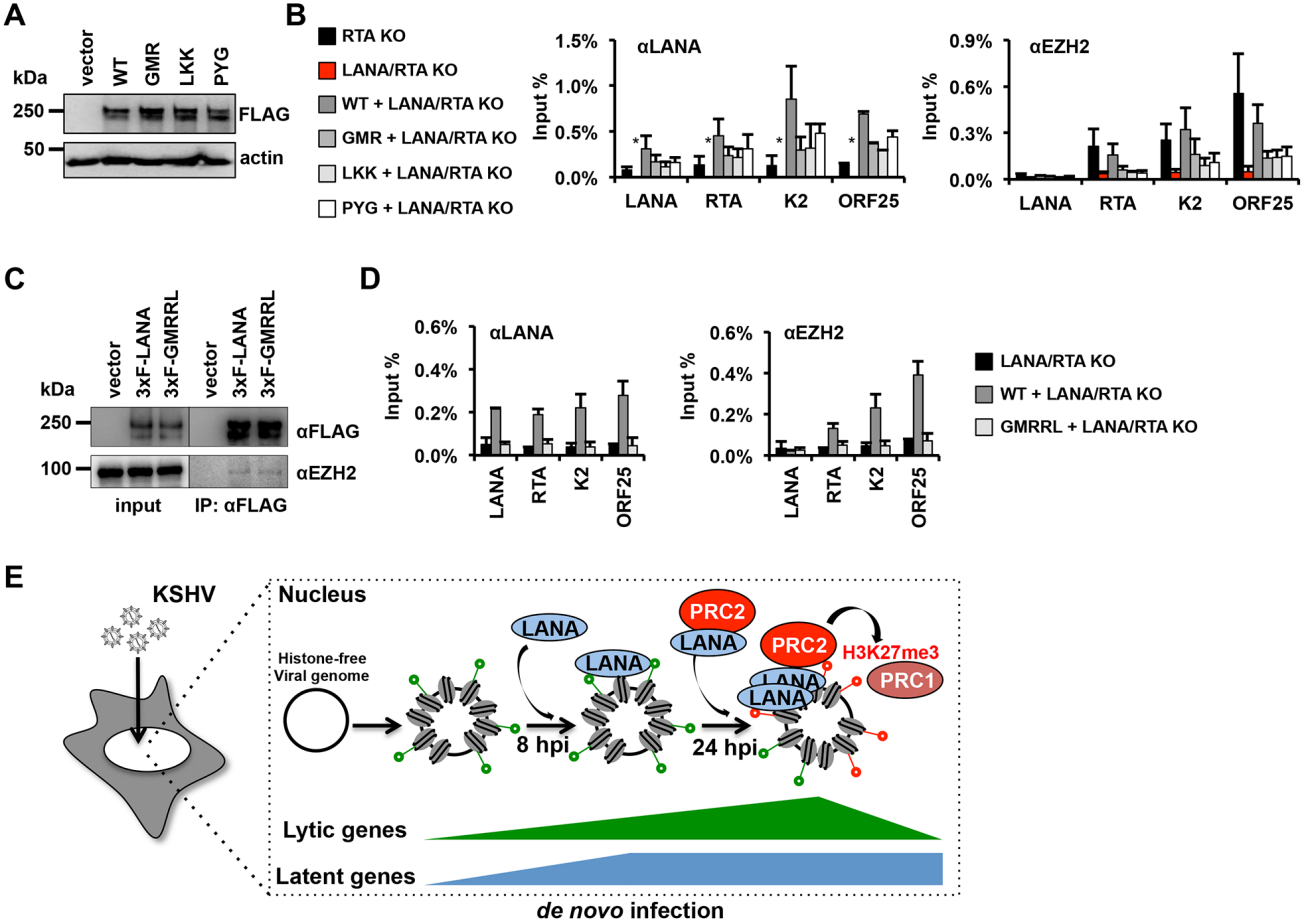
LANA-binding to the lytic KSHV promoters is required for the EZH2 recruitment during *de novo* infection. **(A)** Immunoblot analysis of SLK cells infected with lentiviruses expressing 3xFLAG-tagged WT or mutant LANA using anti-FLAG antibody. **(B)** ChIP assays for the recruitment of LANA (using αLANA antibody) and EZH2 on viral promoters in RTA KO and LANA/RTA dKO KSHV-infected SLK cells as well as in LANA/RTA dKO KSHV-infected SLK cells expressing WT or mutant LANA. **(C)** Total lysates of 293T cells expressing either 3xF-LANA or 3xF-GMRRL mutant LANA were subjected to FLAG immunoprecipitation followed by immunoblotting with anti-FLAG and anti-EZH2 antibodies. Input lysates were included as controls. **(D)** ChIP assays for LANA and EZH2 occupancy on the KSHV promoters during *de novo* LANA/RTA dKO KSHV infection of WT- or GMRRL mutant LANA-expressing SLK cells. **(E)** Model showing LANA-mediated recruitment PRCs onto the KSHV genome during *de novo* infection.

LANA possesses a chromatin-binding domain at its N and C termini as well as a C-terminal DNA-binding domain, both of which are involved in the tethering of the viral genome to the host chromosomes during mitosis [27]. To test which domains of LANA are important for the recruitment of LANA and EZH2 to the KSHV promoters during *de novo* infection, we constructed lentiviruses expressing 3xFLAG-tagged LANA WT or point mutants that are deficient in either DNA or chromatin binding activity, and used them to complement LANA deficiency in LANA/RTA dKO KSHV-infected cells (Fig 7A and 7B). Immunoblot analysis showed comparable expression levels of WT LANA; the N-terminal chromatin-binding mutant, GMR; the C-terminal chromatin-binding mutant, LKK and the DNA-binding mutant, PYG [28] (Fig 7A). Next, we infected WT LANA- or mutant LANA-expressing SLK cells with LANA/RTA dKO KSHV for 72 hours and performed ChIPs for exogenous WT or mutant LANA as well as endogenous EZH2 on KSHV promoters (Fig 7B). RTA KO KSHV-infected SLK cells were included as controls for ChIP assay to show the binding levels of endogenous LANA and EZH2 on the viral promoters (Fig 7B, black color). We found that all the LANA mutants showed reduced binding activity toward the viral promoters compared to WT LANA (Fig 7B). Consequently, the level of EZH2-binding to the viral promoters was also reduced in mutant LANA-expressing cells compared to those in WT LANA-expressing cells. Furthermore, while the GMRRL mutant LANA, deficient in both chromatin- and DNA-binding activities [28], was able to bind endogenous EZH2 as efficiently as WT LANA, it showed a complete loss of its viral promoter-binding activity in LANA/RTA KO KSHV-infected cells and thus, EZH2 was not recruited to viral promoters (Fig 7C and 7D). These results indicate that both the DNA and chromatin binding activities of LANA are required for its binding to the KSHV promoters during *de novo* KSHV infection, which is ultimately crucial for the recruitment of EZH2 onto the lytic viral promoters.

## Discussion

Establishment of latency following *de novo* infection is an essential step for KSHV persistency. Upon infection the viral DNA gets chromatinized in a way that results in the repression of lytic but not the latent genes [11,12]. Our previous study showed that polycomb proteins, which maintain the repression of lytic gene expressions during latency, are recruited onto the KSHV genome after 24 hpi, but the mechanism of their recruitment remained unknown [12]. In this study, we identified the latent KSHV protein LANA to be responsible for the genome-wide recruitment of polycomb proteins onto the viral DNA, a crucial step for the repression of lytic genes following *de novo* infection. Furthermore, we found that despite the co-localization of LANA with both PRCs in the LANA speckles of KSHV-infected cells, LANA interacts only with PRC2 but not PRC1 proteins. This indicates that LANA may not directly recruit PRC1 onto the KSHV genome during *de novo* infection. In fact, we have previously shown that the binding of PRC1 to lytic promoters depends on the PRC2-mediated H3K27me3 pre-deposition on the viral chromatin [12]. Thus, we propose the model wherein as soon as LANA is expressed during *de novo* infection, it binds to the viral episome and recruits PRC2 onto the lytic promoters after 24 hpi. Once H3K27me3 is deposited on the viral chromatin by PRC2, PRC1 is then recruited. Ultimately, this LANA-initiated sequential recruitment of host epigenetic repressors onto lytic promoters results in the inhibition of lytic genes, a critical part of the establishment and maintenance of viral latency (Fig 7E).

Numerous transcription factors and non-coding RNAs (ncRNAs) have been identified to play a role in the recruitment of PRC2 to its target genes on the cellular genome, resulting in either gene-specific or chromosome-wide gene silencing [20]. For instance, X chromosome inactivation in female mammals is regulated by the Xist ncRNA-mediated spreading of PRC2 along the X chromosome [29]. In contrast, PRC2 is simultaneously recruited throughout the KSHV genome following *de novo* infection [11,12]. We have previously demonstrated that the KSHV genome undergoes a temporally-ordered biphasic euchromatin-to-heterochromatin transition following infection prior to the establishment of latency. Between 24–72 hpi, as the level of activating histone marks declined on the KSHV genome, the level of the repressive H3K27me3 histone mark increased concomitantly with the decline of lytic gene expression. Importantly, this transition to heterochromatin was dependent on both PRC 1 and 2 [11,12]. In this report, we identified the latent protein LANA to be responsible for recruiting the polycomb proteins onto the viral episome, which shows a widespread binding on the KSHV genome similar to EZH2 during *de novo* infection. LANA has been shown to possess both sequence specific and promiscuous DNA-binding activity, while it also interacts with histones and numerous transcription and chromatin regulatory factors, which can mediate its genome-wide binding to the KSHV episome [10,30,31,32,33]. It is also known that LANA binds to and is highly enriched on the terminal repeats of KSHV. This contributes to the local LANA concentration in the vicinity of the viral episome in the nucleus, thereby facilitating LANA enrichment on the KSHV genome [8,18,34]. In accordance with our results, LANA has been reported to bind to multiple sites throughout the KSHV genome during latency [13,25,35,36,37,38]. Unlike previous studies that showed the restricted numbers of LANA-binding sites on KSHV genome during latency, our results indicate a broader LANA-binding along KSHV episome during *de novo* infection, suggesting that the differences of LANA-binding are potentially due to the differences of viral lifecyle. Specifically, since the chromatinization of the KSHV genome is a dynamic multi-step process during *de novo* infection, KSHV epigenome and its 3D-chromatin conformation may be not fully developed yet right after infection compared to those during latency conditions, which may affect LANA-binding profiles in the different phases of infection. Furthermore, these differences may be also attributed to the differences of cell lines, ChIP conditions, and ChIPseq analyzing algorithms. Importantly, we included the LANA KO recombinant KSHV as a negative control and the 3xFLAG-LANA recombinant KSHV as a positive control for LANA ChIP assays to examine the widespread binding of LANA on the viral genome during *de novo* infection.

Importantly, we found that a chromatin- and DNA-binding LANA mutant, which lacks the ability to bind to the viral episome could not recruit EZH2 or RING1B to the lytic promoters, indicating an essential role of LANA’s viral genome binding activity in the polycomb recruitment. Since the chromatin-binding or DNA-binding LANA mutants are also defective in the LANA nuclear speckle formation and latent replication of the KSHV genome during mitosis, it is also possible that LANA nuclear speckle formation or LANA-controlled latent replication may also contribute to its PRC2 recruitment [9,39,40,41,42,43]. In addition, our confocal microscopy analysis of infected cells revealed that LANA remained associated with the polycomb proteins through mitosis, indicating a potential role of LANA in transmitting PRCs-regulated heterochromatin to the newly synthesized viral episome during latent replication. Thus, additional studies are required to dissect how various LANA-associated functions can regulate the recruitment of PRC2 onto the viral genome during *de novo* infection.

The highly structured KSHV epigenome characteristic of latency is derived from the sequential formation of distinct chromatin states of the KSHV genome following *de novo* infection [16,17]. However, the factors regulating this process are largely unknown. We have previously shown that the repressive H3K27me3 histone mark is enriched on the viral DNA only after 24 hpi [12]. An elegant study by Gunther and colleagues revealed that the PML factor Sp100 is involved in the regulation of the H3K27me3 level on lytic promoters during *de novo* infection [11]. The spatially and temporally ordered binding of polycomb proteins onto the KSHV genome during *de novo* infection might be similarly regulated by a yet to be identified factor. Our data showed that LANA expression and its binding to viral promoters were substantially lower in the first 24 hpi compared to 72hpi, suggesting that the level of LANA-binding to the viral DNA may not be sufficient for the recruitment of PRC2 within the first 24 hpi. However, we found that both the additional ectopic expression of LANA and the resulting increased level of LANA-binding on the KSHV genome were not sufficient to recruit EZH2 within the first 24 hpi. These results indicate that, in addition to LANA, other factors may be required for the spatially and temporally ordered binding of polycomb proteins onto the KSHV genome during *de novo* infection. Activating histone marks such as H3K4me3, H3K27ac and H3K36me3 and their associated transcription factors can inhibit the binding and activity of PRC2 at specific cellular genomic sites. On the other hand, removal of these histone marks by histone demethylases, deacetylases or chromatin remodeling can allow PRC2-binding [44,45,46]. During the first 24 hours of infection, the KSHV genome acquires transcriptionally active chromatin enriched with the H3K4me3 and H3K27ac. Thus, we hypothesize that the euchromatin-like structure of KSHV may prevent PRC2-binding [11,12]. In fact, the latency locus is devoid of EZH2 even though it has abundant LANA-occupancy and is constantly euchromatin-enriched with the H3K4me3 and H3K27ac. We found that LANA-binding was not affected by the different chromatin states of the viral episome during *de novo* infection, which is consistent with the previous studies showing that LANA binds to both H3K4me3-marked and heterochromatin-rich genomic sites [35,36,47,48]. Alternatively, the differential posttranslational modifications of LANA acquired during the different phases of *de novo* infection may be involved in the temporally ordered binding of EZH2. In addition, LANA has been shown to interact with a number of host epigenetic factors (e.g. KAP1, mSin3, DNMT3a, KDM3A, Tip60, and hSET1), which may also be involved in the timing of PRC2 recruitment to the viral episome during *de novo* infection [10,35,38,49,50,51,52]. Additional studies are required to determine the regulation of the spatially and temporally ordered binding of polycomb proteins onto the KSHV genome during *de novo* infection.

The role of LANA in transcription regulation has been mainly investigated in overexpression conditions and in the absence of KSHV infection. These studies have revealed that LANA regulates both latent and lytic viral gene expression as well as expression of host genes involved in cell cycle and survival signaling pathways [25,26,53,54]. To analyze the transcriptional role of LANA during *de novo* KSHV infection, we determined how the expression of viral and host genes was affected in LANA KO KSHV-infected cells. We found that the lack of LANA resulted in a global upregulation of lytic gene expression in infected cells, which is consistent with the role of LANA in the PRC2-mediated genome-wide gene silencing during *de novo* infection. Importantly, our data also show that the inhibition of lytic gene expression program by LANA was primarily mediated by the repression of RTA expression. LANA has been indicated to bind to and inhibit the RTA promoter during latency, thereby blocking lytic reactivation and maintaining latency [53]. In addition to the viral genome, LANA has also been shown to bind to a large number of transcriptionally active (H3K4me3-marked) promoters and enhancers of the host genome [35,36]. Strikingly, while LANA binds to a number of host promoters in latently infected cells, this binding does not considerably alter expression of these host genes [36]. In agreement with this, we also found weak but global changes of host gene expression in the p53 tumor suppressor and cell cycle regulation pathways in LANA KO KSHV-infected cells compared to those in WT KSHV-infected cells. However, this altered expression of host genes was reversed in LANA/RTA dKO KSHV-infected cells to the levels in WT KSHV-infected cells. Collectively, these data indicate that LANA globally inhibits viral lytic gene expression by repressing RTA expression during *de novo* infection. Otherwise, increased viral lytic gene expression affects the expression of p53 tumor suppressor and cell cycle regulation pathways, which may compromise the establishment of latency.

Accumulating evidence shows that the establishment of latency and long-term persistence of KSHV in B cells and lymphatic endothelial cells is necessary for the development of KSHV-associated cancers. In this study, we identified the KSHV protein that initiates the sequential recruitment of host epigenetic factors onto the KSHV genome during *de novo* infection, which ultimately leads to the genome-wide silencing of lytic genes for the establishment of latency. We envision that many other viral and host factors can also play critical roles in the chromatinization of the KSHV genome prior to the establishment of latency and thus, the identification of these factors should lead to a better understanding of the mechanism of KSHV persistent infection and its associated pathogenesis.

## Materials and Methods

### Cells and antibodies

SLK (NIH AIDS Reagent Program) and 293T (ATCC) cells were maintained in DMEM medium supplemented with 10% FBS, and penicillin/streptomycin (P/S). BCBL-1 (NIH AIDS Reagent Program) was cultured in RPMI medium with 10% FBS and P/S. iSLK cells carrying BAC16 mutants were grown in DMEM medium with 10% FBS, P/S, 1 μg/ml puromycin, 250 μg/ml G418 and 1 mg/ml hygromycin. The origin of iSLK cell line was previously described [55]. The following antibodies were used in ChIPs and immunoblots: anti-histone H3 (Abcam ab1791), anti-H3K27me3 (Active Motif #39155), anti-H3K4me3 (Active Motif #39159), anti-EZH2 (Active Motif #39875), anti-SUZ12 (Active Motif #39357), anti-RING1B (Abcam ab3832), anti-BMI1 (Abcam ab14389), LANA (Advanced Biotechnologies #13-210-100). Anti-SPT5 and anti-CyclinT1 antibodies were purchased from Santa Cruz Biotechnology.

### Generation of recombinant KSHV

The latent gene KO KSHV mutants and 3xFLAG-LANA expressing KSHV were constructed by using en passant mutagenesis on the KSHV BAC clone BAC16 containing the green fluorescence protein (GFP) [56]. All recombinants were verified by pulsed-field gel electrophoresis, PCR and sequencing followed by the generation of iSLK cell lines producing BAC16 mutants as published previously [56]. While the latency locus (Laloc) and the K12 KO mutants were constructed by deleting 9933 base pairs (bps) and 1319 bps from BAC16, respectively, the LANA, v-Cyclin (vCyc) and K13 KO mutants were generated by inserting a stop codon and frameshift mutations close to their translational initiation codons (S1 Table). The BAC16 mutant lacking viral miRNAs (miR) was a gift from the Renne’s lab (University of Florida) [57]. We isolated several clones from each mutant we made, and then tested them by SbfI and NheI restriction enzyme digestions followed by DNA sequencing of the mutation sites. While the digestion patterns of the Laloc and the K12 mutants displayed shifts due to their deletions, those of LANA, vCyc and K13 mutants were indistinguishable from WT BAC16 indicating that there were no detectable genomic rearrangements in the BAC DNAs besides the mutations that we introduced. Next, we established KSHV mutant-carrying iSLK cells, which contain a doxycycline-inducible Replication Transcription Activator (RTA) that is required for the lytic replication of KSHV [55]. Since LANA is essential for the maintenance of KSHV episomes, a complementing iSLK cell line that constitutively expresses LANA was used to produce Laloc or LANA KO viruses. WT and mutant viruses were then used for *de novo* infection of SLK cells, followed by FACS and immunofluorescence (IF) analyses to detect the GFP-positive SLK cells. This showed that the infectivity of KSHV mutants was comparable with that of WT KSHV.

### KSHV production, titration, and infection

To produce KSHV the iSLK cell lines carrying WT or mutant KSHV were treated with 1μg/ml of doxycycline and 1mM sodium butyrate (NaB) for 3.5 days. The virus-containing media were cleared of cell debris by centrifugation at 2000 rpm for 5 minutes, passed through a 0.45μm filter followed by ultracentrifugation using 24,000 rpm for 2.5 hours at 4°C. The virus pellets were resuspended in DMEM and serial dilutions of each KSHV stock were prepared to titer the virus. For this, six-well plates of SLK cells were spin-infected with KSHV (2000 rpm for 45 min at 30°C). At 2 hpi, the media were changed and the numbers of infected cells (based on GFP signal) were determined by flow cytometry (FACS CantoII, BD Bioscience, San Jose, CA) at 24 hpi. Infectious units are expressed as the number of GFP positive cells in each well that was calculated from the total cell numbers per well at the time of analysis. During the titration of KSHV mutants, the amount of KSHV DNA relative to the host genomic DNA was also measured in infected cells. This value was used to adjust the titers of WT and different KSHV mutants in order that equal amounts of KSHV DNA should be used for *de novo* infection. The sequences of DNA oligos used for measuring viral (ORF11 oligos) and host genomic (HS1 oligos) DNAs by qPCR are listed in S2 Table.

### Lentiviral expression of LANA

The pCDHCMV-MCS-EF1-puro lentiviral vector (System Biosciences) was used to express WT and mutant forms of LANA. The LANA mutants were based on studies of Dr. Kenneth Kaye at the Harvard Medical School [28]. Virus-containing supernatants from 293T cells transfected by the lentiviral vector and packaging vectors were collected 60 hours post-transfection, concentrated by centrifugation (24000 rpm, 1.5 hr, 4°C) and used for infection of cells in the presence of 8 μg/ml polybrene. The next day the infected cells were split, and then infected with KSHV the following day.

### ChIP and ChIP-on-chip assays

ChIPs and ChIP-on-chips were performed as published previously [12]. The primer sequences used in ChIP-qPCR are listed in S2 Table. The ChIP graphs display the average of at least two independent experiments. In case of histone H3 modification ChIPs, they were normalized by the histone H3 level in the given genomic sites for their graph presentation. For ChIP-on-chip, 20 μg of chromatin and 2 μg of antibodies while for ChIPs 5–10 μg of chromatin and 0.5–1 μg of antibody were used per ChIP assay. The ChIP-on-chips were performed with our custom-designed Agilent KSHV specific 15-bp tiling microarray, as previously published [12,16]. Briefly, 2 μg of amplified ChIP and input DNAs were submitted to the UCLA Clinical Microarray Core for labeling (Cy3 for input DNA and Cy5 for ChIP DNA), array processing, and microarray scanning. Raw image files were processed with Agilent Feature Extraction software to quantify feature signal intensities and to perform normalization, dye bias correction, and background subtraction. The raw data was pre-processed by blank subtraction (one-step Tukey biweight subtraction) and intra-array (dye-bias) median normalization in order to equalize the ChIP (Cy5) and input (Cy3) DNA channels. Binding events, or enrichments were represented as increases in the ratios of the ChIP to input DNA signal intensities.

### NanoString analysis

This utilizes the simultaneous hybridization of two probes to the target mRNA for detection. One of the probes is the capture probe that can immobilize the target mRNA on a solid surface, while the reporter probes are color-coded allowing the identification of different mRNAs (S3A Fig). We purified total RNAs from WT, LANA KO, and LANA/RTA KO KSHV-infected cells at 72 hpi, and the viral mRNA levels were quantified by our customized panel of KSHV specific NanoString probes at the USC Epigenome Center. The differential viral gene expression of LANA KO virus-infected cells was calculated relative to WT KSHV-infected cells. For normalization the mRNA level of four housekeeping genes and the GFP of BAC16 was used.

### Human gene expression microarray

Total RNA for the microarrays was purified from two biological replicates of uninfected and KSHV-infected SLK cells using Tri reagent (Sigma) and RNeasy Kit (Qiagen) according to the protocols of the manufacturers. The human gene expression profiling was performed with the Affymetrix Human Genome U133 Plus 2.0 Array at the UCLA Clinical Microarray Core. The raw expression data are available from the GEO database (accession number GSE78282). All microarray data analyses were performed by Partek Genomics Suite 6.6 unless otherwise indicated. The raw expression values were first normalized together with Robust Multichip Analysis (RMA). The RMA normalized data file containing all probes and their expression values with their gene IDs can be found in S3 Table. After performing microarray quality control, differential gene expression analysis was performed. For this, we employed hierarchical clustering using the average of the probe intensities of the duplicate biological experimental array datasets, which passed the threshold of 1.5 fold difference between uninfected and infected samples. The list of differentially expressed genes is in S3 Table. Gene Set Enrichment Analysis (GSEA) between the wild type and LANA KO virus-infected samples was performed following guidelines as detailed on the GSEA website [58]. Using 1000 permutations of the GSEA hallmark gene sets and a false discovery rate (FDR) of less than 0.05 was accepted as highly significant, which was highlighted in Fig 3C. S4 Table contains the full statistical GSEA analysis.

### Immunofluorescence microscopy

The uninfected and infected cells were grown on glass coverslips. The cells were washed with PBS, fixed with 4% paraformaldehyde for 10 minutes at room temperature, and then permeabilized with 0.5% Triton X-100 for 5 minutes and stored in PBS containing 0.2% Tween 20 (PBST) at 4°C. Prior to the immunofluorescent staining, the samples were incubated in blocking solution (10% donkey serum, 0.2% Tween 20, 0.2% Fish Skin gelatin in PBS) for 30 minutes. The antibodies were diluted in PBST. The primary antibodies that were used are anti-LANA (1/3000 dilution), anti-FLAG (1/100 for *de novo* infected cells and 1/1000 dilution for latently infected cells), anti-EZH2, anti-SUZ12, anti-BMI, anti-RING1B, anti-CYCT1, and anti-SPT5 (1/100 dilution). Secondary antibodies (goat anti-rat 633, goat anti-rabbit 568, and donkey anti-mouse 647) were used in 1/500 dilution. Primary antibody staining was performed for 2 hours followed by three times 5 minutes wash with PBST. This was followed by 45 minutes of incubation with secondary antibodies then three times 5 minutes wash with PBST. Thereafter, ProLong Diamond Antifade Mountant with DAPI reagent (Thermo Fisher Scientific) was applied and the coverslips were layered onto microscope slides. The samples were analyzed by confocal microscopy using 60x objective and the images were processed, analyzed, merged, and quantified with ImageJ software.

### Gel filtration chromatography

Nuclear extracts derived from 10^8^ of iSLK and iSLKBAC16 cells were fractionated on a GE Superose 6 GL column using a BioLogic DuoFlow Medium-Pressure Chromatography system (Bio-Rad). The gel filtration running buffer contains 40 mM Tris-HCl (pH 7.5), 300 mM NaCl, 40mM NaF, 40 mM β-glycerophosphate, 1 mM sodium orthovanadate (Na_3_VO_4_), 5mM Metabisulphite sodium salt, 10mM Benzamidine, 2mM EDTA, 1mM EGTA, and 20% glycerol. The chromatography system was operated with 0.2 ml/min flow rate and 217 psi maximum pressure. The collected fractions were analyzed by immunoblots. The molecular mass of eluted proteins was estimated by pre-running of standard gel filtration markers (Sigma, MWGF1000).

### Co-immunoprecipitation assay

Whole cell lysates (WCL) were prepared from transfected 293T cells or *de novo* KSHV-infected iSLK cells using 0.5% NP40 lysis buffer [10mM Tris-HCL (pH 8.0), 100mM NaCl, 1mM EDTA, 0.5% NP-40] containing protease inhibitor cocktail (Roche). Lysates were precleared with sepharose beads for 2 h at 4°C. WCL were incubated with the indicated antibodies overnight, followed by further incubation with protein A/G-conjugated beads for 2 h at 4°C. Immunoprecipitation complexes were washed three times with 0.5% NP40 lysis buffer. SDS-PAGE gel was transferred onto PVDF membrane followed by blocking in 5% non-fat milk and then immunoblotting with the indicated antibodies.

## Supporting Information

S1 Table DNA sequences of the latent gene KO KSHV mutants. (XLSX)

S2 Table Primer sequences used in this study. (XLSX)

S3 Table Differential cellular gene expression upon KSHV infection. (XLSX)

S4 Table Comparison of the cellular gene expression of WT and LANA KO KSHV infected cells by GSEA analysis. (XLSX)

S1 Fig Construction and production of infectious latent gene KO KSHV mutants. **(A)** Pulse-field gel electrophoresis of Sbf I- and Nhe I-digested BAC DNAs. MW1 and MW2 indicate molecular weight markers. **(B)** Schematic depiction of the LANA protein showing the acidic central repeat regions (CR1-3), the nuclear localization signal (NLS), and the position of the STOP codon insertion. **(C)** Immunoblot analysis of LANA expression in WT and LANA KO KSHV-infected SLK cells. **(D)** Detection of GFP-marked infected cells. **(E)** SLK cells were infected with the same titer of WT and LANA KO KSHV at day 0, followed by splitting the cells at a 1:4 ratio every other day for 29 days. The number of infected cells was monitored by flow cytometry to detect the GFP-positivity (%). Note: GFP was not detected in LANA KO KSHV-infected cells from day 11. **(F)** GFP-positive cells infected with WT and LANA KO KSHV were detected by fluorescence analysis at different time points of post-infection.
(TIF)

S2 Fig Effect of the LANA deletion on the enrichment of chromatin regulatory factors on KSHV promoters during *de novo* infection. **(A)** ChIP assays for the enrichment of CTCF and RAD21 chromatin architecture regulatory proteins on viral promoters in KSHV-infected SLK cells at 72 hpi. RTApr and RTAint indicate the promoter and the intron region of RTA, respectively. HS1 and Neg cellular genomic sites were used as controls. **(B)** Immunoblot analysis of LANA protein levels in shLANA-treated SLK cells. Four different shRNAs were tested for depletion of LANA expression. **(C)** Control or shLANA_2-treated SLK cells were infected with either WT or RTA KO KSHV, followed by ChIP assays for the indicated histone marks and the PRC1 factor RING1B on the KSHV promoters at 72 hpi.
(TIF)

S3 Fig Analysis of viral and host gene expressions in WT or LANA KO KSHV-infected cells. **(A)** Schematic depiction of the components of the NanoString assay. **(B)** Analysis of viral gene expression in WT and mutant KSHV-infected cells at 72 hpi using gene specific qPCR. **(C)** Analysis of viral gene expression in shLANA-treated KSHV-infected cells at 72 hpi using gene specific qPCR. **(D)** Differential host gene expression between WT and LANA KO KSHV-infected cells. The number of microarray probes and their corresponding number of genes are indicated in parentheses. Examples from each cluster are indicated below the diagrams. **(E)** Analysis of host gene expression in WT and mutant KSHV-infected cells at 72 hpi using gene specific qPCR.
(TIF)

S4 Fig LANA and KSHV DNA complex formation during *de novo* KSHV infection. **(A)** Time course ChIP analysis of LANA-binding on viral promoters during *de novo* WT KSHV infection. ChIPs in LANA/RTA dKO KSHV-infected cells were performed at 72 hpi. Promoters of the cellular genes ACT, MYT1, and HTF6 were used as controls. **(B)** Confocal microscopic analysis of LANA expression in iSLK cells infected with BAC16-3xF-LANA. Anti-FLAG antibody was used for detection of LANA (red) and GFP indicates the infected cells. Long-term KSHV latently infected iSLKBAC16 cells were used as controls. Zoom-in pictures of the nuclei indicated by white arrow are shown on the right.
(TIF)

S5 Fig Co-localization of LANA with PRC2 factors in KSHV-infected cells. KSHV-infected iSLK and TIME (TIMEBAC16) cells were subjected to confocal microscopy to analyze the co-localization of LANA (green, false color) with SUZ12 or EZH2 (red) during *de novo* infection (C, D and K), latency (E, F and L-O), and reactivation (G and H). Uninfected iSLK cells were used as controls (A, B and I, J). For reactivation, iSLKBAC16 cells were induced by 1 μg/ml of doxycycline and 1 mM of sodium butyrate for 24 or 48 hours. Panels B, F, J and M show mitotic chromosomes. Representative LANA puncta were connected by white marked lines and the co-localization of LANA with SUZ12 or EZH2 was measured using the image processing program ImageJ.
(TIF)
