# Supplementary figures and images for: LANA-Mediated Recruitment of Host Polycomb Repressive Complexes onto the KSHV Genome during De Novo Infection
Source: PLoS Pathog. 2016 Sep 8;12(9):e1005878. doi: 10.1371/journal.ppat.1005878 (PMC5015872; doi:10.1371/journal.ppat.1005878)

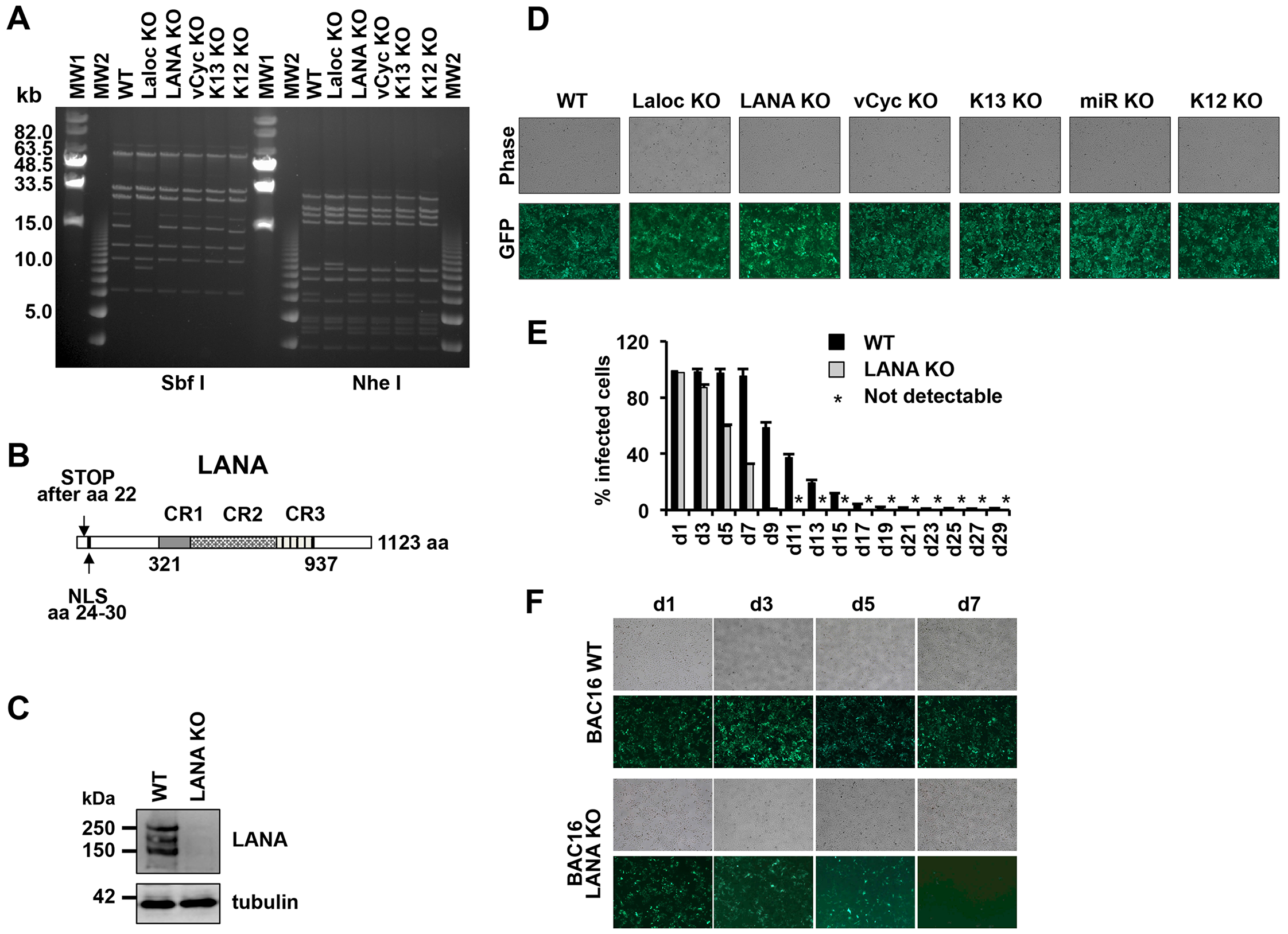

Supplement: S1 Fig — (A) Pulse-field gel electrophoresis of Sbf I- and Nhe I-digested BAC DNAs. MW1 and MW2 indicate molecular weight markers. (B) Schematic depiction of the LANA protein showing the acidic central repeat regions (CR1-3), the nuclear localization signal (NLS), and the position of the STOP codon insertion. (C) Immunoblot analysis of LANA expression in WT and LANA KO KSHV-infected SLK cells. (D) Detection of GFP-marked infected cells. (E) SLK cells were infected with the same titer of WT and LANA KO KSHV at day 0, followed by splitting the cells at a 1:4 ratio every other day for 29 days. The number of infected cells was monitored by flow cytometry to detect the GFP-positivity (%). Note: GFP was not detected in LANA KO KSHV-infected cells from day 11. (F) GFP-positive cells infected with WT and LANA KO KSHV were detected by fluorescence analysis at different time points of post-infection. (TIF) [file ppat.1005878.s005.tif]

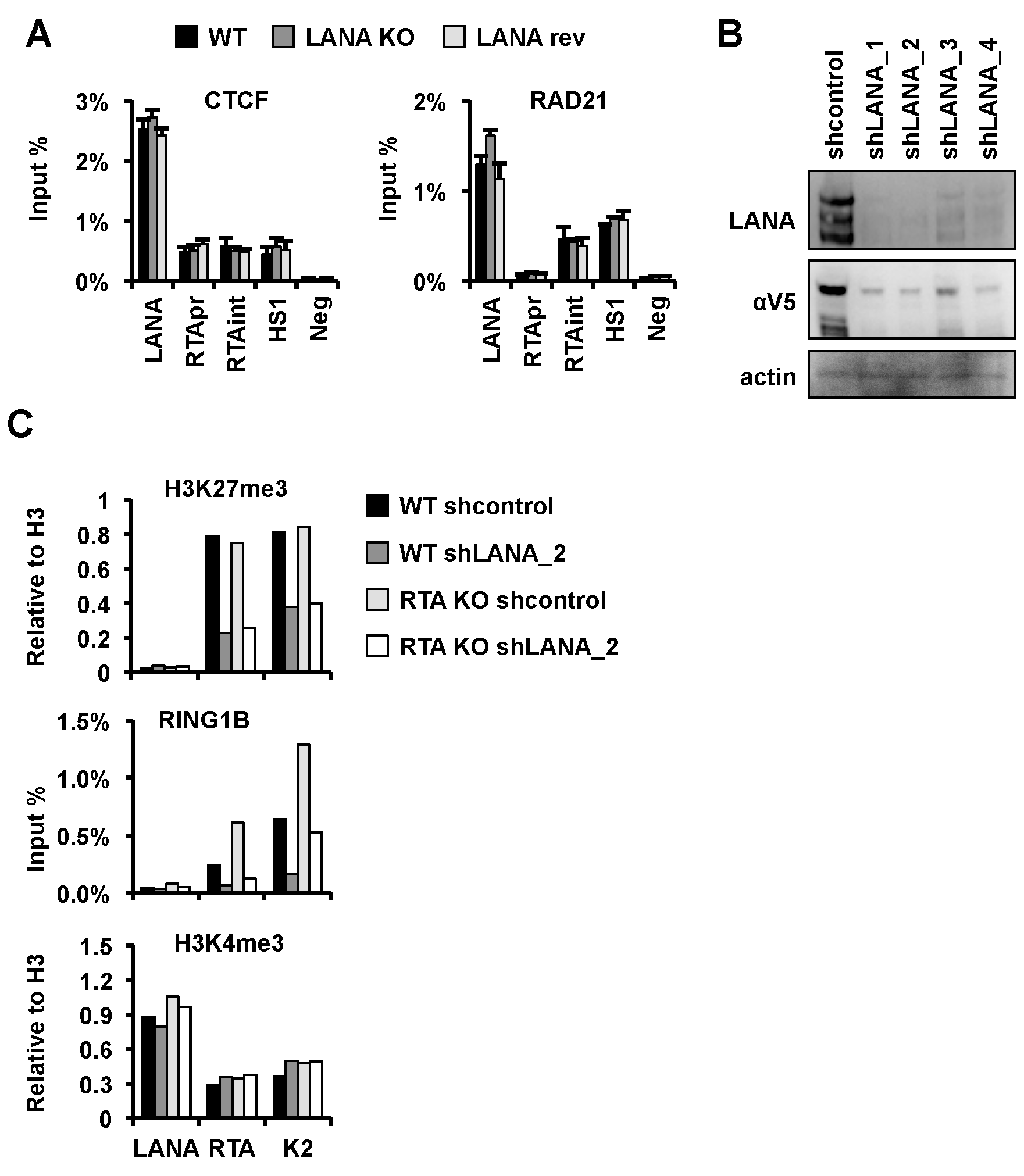

Supplement: S2 Fig — (A) ChIP assays for the enrichment of CTCF and RAD21 chromatin architecture regulatory proteins on viral promoters in KSHV-infected SLK cells at 72 hpi. RTApr and RTAint indicate the promoter and the intron region of RTA, respectively. HS1 and Neg cellular genomic sites were used as controls. (B) Immunoblot analysis of LANA protein levels in shLANA-treated SLK cells. Four different shRNAs were tested for depletion of LANA expression. (C) Control or shLANA_2-treated SLK cells were infected with either WT or RTA KO KSHV, followed by ChIP assays for the indicated histone marks and the PRC1 factor RING1B on the KSHV promoters at 72 hpi. (TIF) [file ppat.1005878.s006.tif]

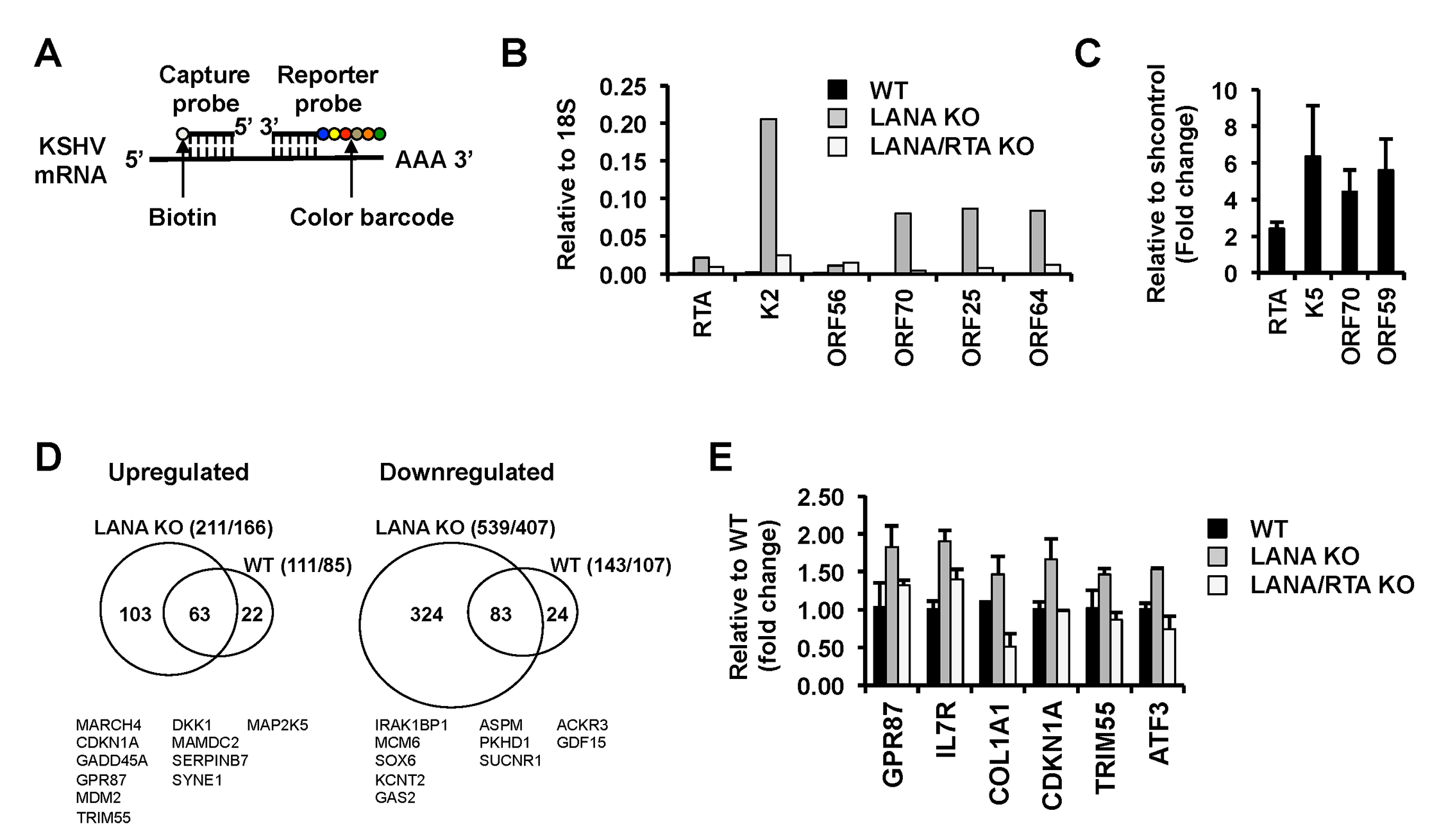

Supplement: S3 Fig — (A) Schematic depiction of the components of the NanoString assay. (B) Analysis of viral gene expression in WT and mutant KSHV-infected cells at 72 hpi using gene specific qPCR. (C) Analysis of viral gene expression in shLANA-treated KSHV-infected cells at 72 hpi using gene specific qPCR. (D) Differential host gene expression between WT and LANA KO KSHV-infected cells. The number of microarray probes and their corresponding number of genes are indicated in parentheses. Examples from each cluster are indicated below the diagrams. (E) Analysis of host gene expression in WT and mutant KSHV-infected cells at 72 hpi using gene specific qPCR. (TIF) [file ppat.1005878.s007.tif]

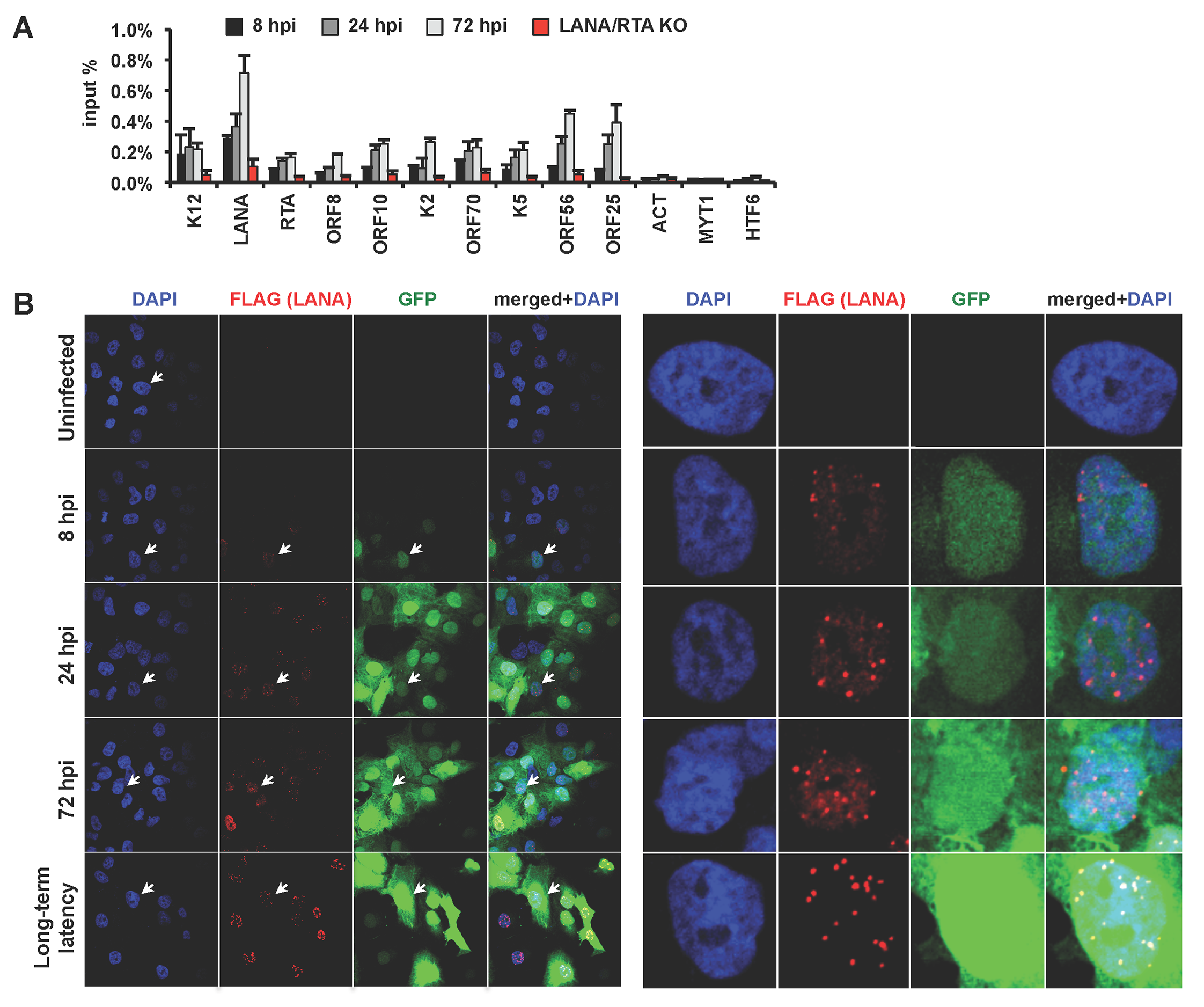

Supplement: S4 Fig — (A) Time course ChIP analysis of LANA-binding on viral promoters during de novo WT KSHV infection. ChIPs in LANA/RTA dKO KSHV-infected cells were performed at 72 hpi. Promoters of the cellular genes ACT, MYT1, and HTF6 were used as controls. (B) Confocal microscopic analysis of LANA expression in iSLK cells infected with BAC16-3xF-LANA. Anti-FLAG antibody was used for detection of LANA (red) and GFP indicates the infected cells. Long-term KSHV latently infected iSLKBAC16 cells were used as controls. Zoom-in pictures of the nuclei indicated by white arrow are shown on the right. (TIF) [file ppat.1005878.s008.tif]

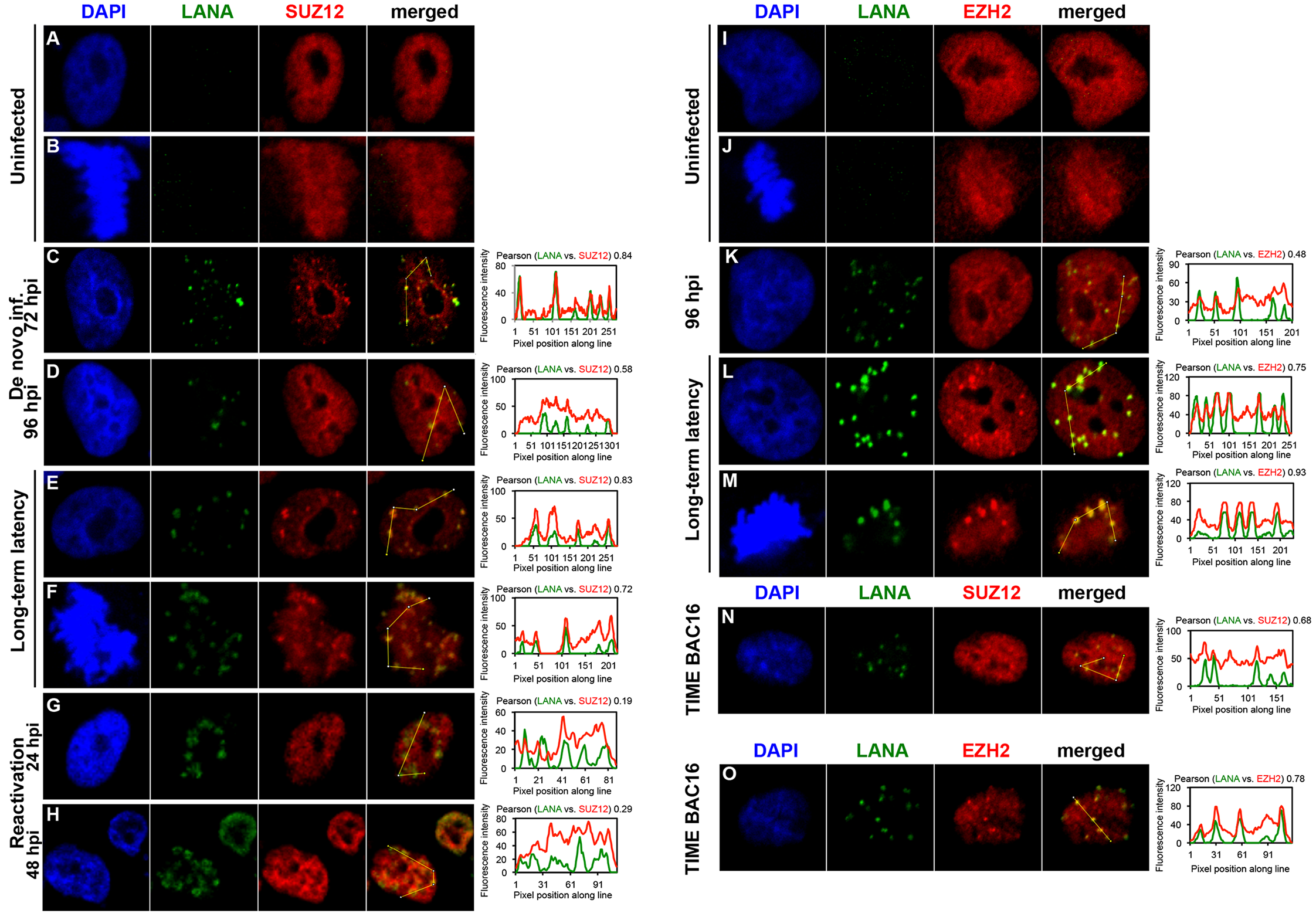

Supplement: S5 Fig — KSHV-infected iSLK and TIME (TIMEBAC16) cells were subjected to confocal microscopy to analyze the co-localization of LANA (green, false color) with SUZ12 or EZH2 (red) during de novo infection (C, D and K), latency (E, F and L-O), and reactivation (G and H). Uninfected iSLK cells were used as controls (A, B and I, J). For reactivation, iSLKBAC16 cells were induced by 1 μg/ml of doxycycline and 1 mM of sodium butyrate for 24 or 48 hours. Panels B, F, J and M show mitotic chromosomes. Representative LANA puncta were connected by white marked lines and the co-localization of LANA with SUZ12 or EZH2 was measured using the image processing program ImageJ. (TIF) [file ppat.1005878.s009.tif]
